# Using Hashimoto thyroiditis as gold standard to determine the upper limit value of thyroid stimulating hormone in a Chinese cohort

**DOI:** 10.1186/s12902-016-0137-3

**Published:** 2016-11-06

**Authors:** Yu Li, Dong-Ning Chen, Jing Cui, Zhong Xin, Guang-Ran Yang, Ming-Jia Niu, Jin-Kui Yang

**Affiliations:** 1 Physical Examination Department, Beijing Tongren Hospital, Capital Medical University, Beijing, 100730 China; 2 Department of Endocrinology, Beijing Key Laboratory of Diabetes Research and Care, Beijing Tongren Hospital, Capital Medical University, Beijing, 100730 China; 3 Department of Endocrinology, First Hospital of Qinghuangdao, Qinghuangdao, 066000 China

## Abstract

**Background:**

Subclinical hypothyroidism, commonly caused by Hashimoto thyroiditis (HT), is a risk factor for cardiovascular diseases. This disorder is defined as merely having elevated serum thyroid stimulating hormone (TSH) levels. However, the upper limit of reference range for TSH is debated recently. This study was to determine the cutoff value for the upper normal limit of TSH in a cohort using the prevalence of Hashimoto thyroiditis as “gold” calibration standard.

**Methods:**

The research population was medical staff of 2856 individuals who took part in health examination annually. Serum free triiodothyronine (FT3), free thyroxine (FT4), TSH, thyroid peroxidase antibody (TPAb), thyroglobulin antibody (TGAb) and other biochemistry parameters were tested. Meanwhile, thyroid ultrasound examination was performed. The diagnosis of HT was based on presence of thyroid antibodies (TPAb and TGAb) and abnormalities of thyroid ultrasound examination. We used two different methods to estimate the cutoff point of TSH based on the prevalence of HT.

**Results:**

Joinpoint regression showed the prevalence of HT increased significantly at the ninth decile of TSH value corresponding to 2.9 mU/L. ROC curve showed a TSH cutoff value of 2.6 mU/L with the maximized sensitivity and specificity in identifying HT. Using the newly defined cutoff value of TSH can detect patients with hyperlipidemia more efficiently, which may indicate our approach to define the upper limit of TSH can make more sense from the clinical point of view.

**Conclusions:**

A significant increase in the prevalence of HT occurred among individuals with a TSH of 2.6–2.9 mU/L made it possible to determine the cutoff value of normal upper limit of TSH.

## Background

Patients with subclinical hypothyroidism have normal level of serum thyroid hormones (T4, T3, FT3, FT4) and elevated TSH. These patienshave a higher incidence of lipid abnormalities, coronary heart disease, psychiatric disorders and pregnancy complications [1–9], although their clinical symptoms are very mild. Proper screening and treatment of these patients may help to improve the adverse outcome of the involving diseases. Therefore, to define the upper limit of TSH precisely had an important role in detecting patients who had mild thyroid dysfunction and might benefit from early intervention.

The upper limit of reference range for “normal” TSH has been the focus of debate in the recent decade. Some authors insisted on the conventional value of 4.0–5.0 mU/L as the upper limit of normal thyroid stimulating hormone (TSH) [10], others suggested it narrowed to 2.5–3.0 mU/L [11]. As mentioned in the National Academy of Clinical Biochemistry (NACB) guideline, more than 95 % of normal individuals had TSH below 2.5 mU/L. There were even data showing that African-Americans had very low incidence of HT with a mean TSH level of 1.18 mU/L This value maybe the “true normal” upper limit for TSH, because African-Americans have very low prevalence of Hashimoto thyroiditis (HT) to elevate TSH [11].

Another question was put forward that which was the true set point of normal upper limit. Which centile (90, 95 or 99th) should be the real normal limit? The approximate questions related to glycemic threshold for diagnosing diabetes were already answered by using diabetic retinopathy (DR) as “gold” calibration standard [12]. Referring to that and based on the correlation between HT (as end-point) and TSH value, we tried using the prevalence of HT as the calibration standard to determine the upper limit of TSH.

## Methods

### Research population

A total of 2856 medical staffs with age between 20 and 60 years old participated in a health examination in the year of 2013 were included in our research. Questionnaires related to medication history and other health-related behaviors were completed beforehand. Blood sampling was performed in the morning after eight hours of fasting, followed by the detection of height, weight, waist-to-hip ratio (WHR), blood pressure and physical examinations.

The study was conducted with the approval of the Ethics Committee of Beijing Tongren Hospital, Capital Medical University.

### Laboratory assessments

Blood samples were collected for testing thyroid function including serum free triiodothyronine (FT3), free thyroxine (FT4) and TSH, thyroid peroxidase antibody (TPAb), thyroglobulin antibody (TGAb) and other biochemistry parameters including total cholesterol (TC), triglyceride (TG), low density lipoprotein cholesterol (LDL),, fasting plasma glucose (FPG), uric acid (UA), alanine aminotransferase (ALT), aspartate aminotransferase (AST), creatinine (Cr) and carbon dioxide combining power (CO_2_CP). Appliance for testing the above mentioned biochemistry factors were Immunoassay Systems (Beckman Coulter, UniCel DXI800).

### The diagnostic criteria for HT

The diagnosis of HT was established by a combination of presence of thyroid antibodies (TPAb and TGAb) and abnormalities of thyroid sonogram including reduced echo or diffused heterogeneity echo of thyroid with or without nodularity [8]. Ultrasound examination of thyroid was performed by four experienced professional ultrasound physicians. Consistent diagnosis standard was used, including description for the size, nodules and echo of the thyroid.

### Framingham score

Framingham score (FS), a 10-year estimated risk of CHD, was calculated by the equation including: age, gender, blood pressure, smoking status and cholesterol levels [13]. FS equaling to or over 9 was considered to be a predictor for the higher CHD risk of over 5 %.

### Statistical analysis

Unpaired *t*-test, Mann-Whitney *U* test or Pearson Chi-square test was used to compare the difference between cohorts with or without HT. We used two different methods to estimate the cutoff point of TSH based on the prevalence of HT: Joinpoint regression [14] and ROC curve by maximizing the sensitivity and specificity. All statistical analyses were conducted with the software package SPSS version 13 for windows, and ljr package of R software (http://www.r-project.org). A two-sided *p* value of less than 0.05 was considered to be statistically significant.

## Results

### Characteristics of the observed cohort

By the exclusion standard, those with abnormal thyroid function (FT3 and FT4 out of the normal range), being pregnant, taking thyroid related medicine, accepted thyroid operation and patients with personal history of autoimmune disorders were excluded. A total number of 2856 individuals were included in the research, with the average age of 36.0. The proportion of women (75 %) was higher than men (25 %), which was consistent with the gender characteristic of the medical staff in China.

The research population was further divided into HT and non-HT groups. According to the diagnosis standard, 187 (7 %) in the observed cohort had HT (Table 1), which comprised of 14 males and 173 females. The prevalence of HT in females (8 %) was 4 times higher than in males (2 %).

**Table 1:** Characteristics of observed cohort divided by the participants with Hashimoto thyroiditis (HT) or without HT (non-HT)

	Non HT	HT	*P*
No	2669	187	---
Age (year)	35.90 (9.15)	36.99 (9.25)	0.117
Sex (M/F)	704/1965	14/173	<0.001**
TSH (μIU/l)	1.83 (0.01–26.36)	2.66 (0.01–39.96)	0.001*
Free triiodothyronine (pmol/l)	4.96 (0.68)	4.95 (0.92)	0.92
Free thyroid hormone (pmol/l)	10.58 (1.72)	10.36 (1.92)	0.12
BMI (kg/m2)	23.23 (3.61)	23.33 (3.31)	0.69
WHR	0.81 (0.07)	0.80 (0.06)	0.14
Systolic blood pressure (mmHg)	114.15 (13.95)	113.66 (13.61)	0.64
Diastolic blood pressure (mmHg)	73.22 (9.39)	72.87 (8.71)	0.60
Alanine aminotransferase (IU/l)	24.26 (17.40)	22.84 (12.04)	0.13
Aspartate aminotransferase (IU/l)	26.53 (9.68)	26.41 (7.73)	0.84
Blood urea nitrogen (mmol/l)	3.80 (1.05)	3.72 (0.99)	0.28
Creatinine (μmol/l)	65.54 (13.99)	60.09 (9.68)	<0.001*
Total cholesterol (mmol/l)	4.64 (0.84)	4.65 (0.83)	0.87
Triglycerides (mmol/l)	1.10 (0.91)	1.04 (0.73)	0.34
LDL cholesterol (mmol/l)	2.89 (0.89)	2.88 (0.74)	0.87
HDL cholesterol (mmol/l)	1.45 (0.39)	1.47 (0.34)	0.45
Fasting plasma glucose (mmol/L)	5.33 (0.94)	5.24 (0.50)	0.16
Uric acid (mmol/L)	278.90 (74.60)	264.48 (67.47)	**0.010**

Values are mean(SD) or median (range). Comparison between any two groups by unpaired *t*-test, Mann-Whitney *U* test* or Pearson Chi-square test**. Data in bold are statistically significant

No significant differences between the HT and non-HT groups were found in age, BMI, WHR, blood pressure or biochemical parameters concerning liver function, blood glucose and lipid. However, serum creatinine and uric acid were lower in HT group, which may be related to the high prevalence of HT in females, because females have relatively low level of serum creatinine and uric acid than males.

### The distribution of TSH in the total population and subgroups (HT and non-HT group)

**Fig. 1:**
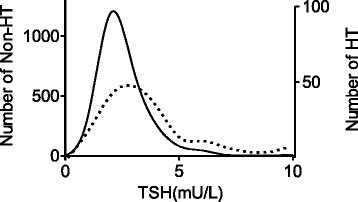
Distribution of thyroid stimulating hormone (TSH) in both Hashimoto thyroiditis (HT) (− − −) and non-HT (–––– –––– –––– –––– –––––––– –––––––––––––––––––––––––––––––––––––––––––––––––) participants. Curves were smoothed by the GraphPad Prism 5 software

Although exclusion standard was used in our research to get a relatively homogeneous cohort without overt thyroid diseases, the distribution of TSH still showed asymmetrical curve with extension to the right side. We then separated HT from the whole population, and the TSH distribution of HT and non-HT groups was described in Fig. 1. Non-HT group showed a more likely Gaussian curve, and the HT group showed an uneven curve with more rightward trend representing higher TSH values. The different centiles of TSH in both groups were listed in Table 2. By ANOVA analysis, there was no statistically significant influence of age to the upper limit of TSH in our research population (*P* = 0. 5).

**Table 2:** Serum TSH levels (mU/L) in total population and subgroups categorized by age

		Percentile				
Age (yr)	Sample No.	2. 5th	5th	Median	95th	97.5th
A. total						
19–29	845	0.53	0.73	1.80	4.45	5.50
30–39	1060	0.51	0.73	1.85	4.42	4.98
40–49	662	0.61	0.73	1.94	4.99	5.61
50–59	289	0.65	0.70	1.92	5.45	7.49
B. non-HT						
19–29	799	0.56	0.75	1.79	4.33	5.19
30–39	989	0.52	0.74	1,82	4.06	4.62
40–49	616	0.62	0.73	1.91	4.31	5.26
50–59	265	0.65	0.70	1.86	4.58	5.67
C. HT						
19–29	46	0.37	0.74	2.33	8.48	21.67
30–39	71	0.02	0.46	2.43	9.50	18.86
40–49	46	0.03	0.33	3.29	8.34	24.26
50–59	24	0.56	0.73	3.17	14.77	15.50

### The prevalence of HT corresponding to each decile of TSH value

**Fig. 2:**
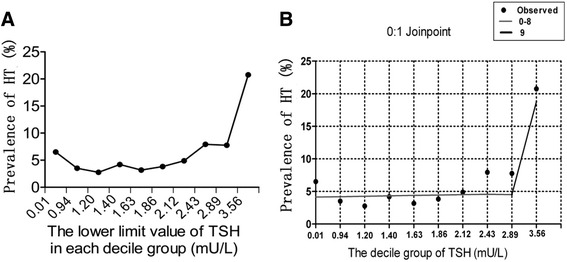
**a** The prevalence of HT corresponding to each decile of TSH value (Each point in the chart corresponded to the number labeled in X axis. 2.89 is the cutoff value of TSH revealed by joinpoint analysis). **b** The joinpoint regression analysis showed only one joinpoint was found. This joinpoint presented in the ninth decile group, and the lower limit value of this group of 2.89 was taken as the cutoff

A curve was drawn to describe the correlation between HT and TSH value (Fig. 2). Each decile of TSH value was marked in the X-axis, meanwhile the proportion of participants with HT between each decile of TSH was shown in Y-axis. The curve was relatively flat when TSH <2.9 mU/L. Whereas the curve showed a sharp increase at the ninth decile of TSH (corresponding to TSH≧2.9 mU/L) The prevalence of HT in the ninth decile was 21 %, while that in the eighth decile was 8 %. Regression analysis showed a good Joinpoint at the ninth decile (*P* = 0.0012). Logistic regression models adjusted for sex, age, BMI, FT4, systolic blood pressure and biochemical parameters also confirmed a statistically significant difference for the prevalence of HT below vs. above the cutoff point for TSH value (OR 3.07 [95 % CI: 2.24–4.21]; *P* = 0.000).

### ROC curve describing the cutoff value of TSH in detecting participants with HT

**Fig. 3:**
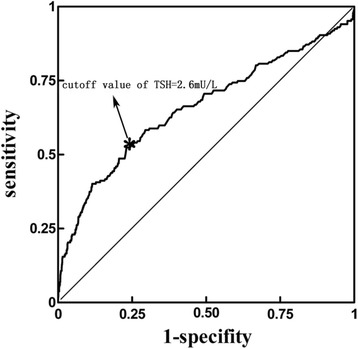
ROC curve describing the cutoff value of TSH in detecting participants with HT. When maximizing the sensitivity and specificity, the cutoff value of TSH equaled to 2.6 mU/L, with the sensitivity of 52 % and the specificity of 77 %

When maximizing the sensitivity and specificity, the cutoff value of TSH equaled to 2.6 mU/L, with the sensitivity of 52 % and the specificity of 77 %. The area under the ROC curve was 66 % (95 % CI: 61.01–70.51) (Fig. 3). The proportion of participants with HT was 4 % when TSH was below 2.6 mU/L. As compared, when TSH was above 2.6, the proportion of participants with HT was 14 % (*P* = 0.000 by Fisher’s exact test).

### Comparison of metabolism-related parameters between groups divided by different cutoff values of TSH

As shown in Table 3, the cutoff value of 2.9 mU/L (based on the Joinpoint regression), and 2.6 mU/L (based on the ROC curve analysis), and 4.5 mU/L (conventionally accepted cutoff value) were used to categorize the population. All three grouping methods showed a higher probability of hyperlipidemia and a higher Framingham Score in participants with TSH above the cutoff point, and a relatively younger age in participants with TSH below the cutoff point. As compared to using 4.5 mU/L as the cutoff point, using 2.6 mU/L or 2.9 mU/L as the cutoff value of TSH can detect more patients with abnormal TC and LDL. That may tell us it’s more effective to apply the lowered cutoff value of TSH for detecting patients with dyslipidemia.

**Table 3:** Comparison of metabolism related parameters between groups divided by cutoff values of TSH based on different method (using Joinpoint regression, ROC curve and conventional accepted value respectively)

Derivation of cutoff values	Group	No	Age (mean(SD))	BMI ≧24	Hyper- TC	Hyper-LDL-c	Hyper-TG	Hyper- FPG	FS≧9
Joinpoint regression	TSH < 2.9	2284	35.7 (9.1)	37 %	22 %	23 %	14 %	7 %	20 %
	TSH≧2.9	572	36.9 (9.5)	35 %	27 %	27 %	18 %	8 %	23 %
	*P*		0.009	0.49	0.021*	0.07	0.030*	0.46	0.18
ROC curve	TSH < 2.6	2138	35.7 (9.1)	37 %	22 %	23 %	14 %	7 %	20 %
	TSH≧2.6	718	36.8 (9.5)	36 %	28 %	27 %	17 %	8 %	23 %
	*P*		0.008	0.44	<0.001*	0.007*	0.024*	0.21	0.034*
Conventional accepted	TSH < 4.5	2714	35.9 (9.1)	37 %	23 %	24 %	15 %	7 %	20 %
	TSH≧4.5	142	37.7 (10.2)	38 %	26 %	26 %	20 %	9 %	31 %
	*P*		0.046	0.77	0.008*	0.50	0.09	0.39	0.003*

Values are means(SD) or probability. Comparison between any two groups by unpaired *t*-test, or Pearson Chi-square test^*^. Data in bold are statistically significant

*FS* Framingham score, *LDL-c* low-density lipoprotein cholesterol, *TG* triglyceride, *FPG* fasting plasma glucose

## Discussion

To get a more convincing reference range of TSH, previous researchers used a more stringent standard for screening the reference population proposed by the NHANES III study [2], and more sensitive assays were used to measure TSH of those reference individuals. The upper limit of TSH was lowered to 3.6–3.7 mU/L [3, 15]. It was believed that the skewing right tail of the TSH distribution curve was caused by the inclusion of those with occult thyroid dysfunctions most representatively as HT. Besides, other factors such as gender and age are also critical to the reference range of TSH [16]. In this study, age does not serve as an influence factor to the upper limit of TSH. The reason may be the research population was a homogeneously young cohort. Besides, It is not an iodine deficient area in Beijing. Therefore, the daily iodine intake and total body iodine status should not be a major factor to influence TSH value in our research population. We aimed to clear up the confounding influence of those individuals with HT to determine the cutoff point of TSH in our research population.

If we defined the upper limit of normal TSH as the 95th centile as adopted by previous researchers, the value would be 4.2, which could be comparable to most publications and more approximate to the traditional opinions insisting on 4.0–5.0 mU/L as the upper limit of normal TSH.

However, there were still considerable proportion of people with occult subclinical thyroid disease (mostly HT) within the upper part of the normal TSH range. Whereas we expect the normal range as a tool to screen out abnormal individuals. We tried an alternative approach to exclude the influence to TSH value confounded by individuals with HT. Based on the fact that TSH and the prevalence of HT was well correlated, we used “the prevalence of HT” as a checkout factor to determine the cutoff upper limit of TSH. Mimicking the diagnostic methodology in determining glycemic thresholds using DR as the checkout factor, we drew a curve describing the correlation between the prevalence of HT and TSH value. From this curve we got a turning point at TSH 2.9 mU/L by Joinpoint regression analysis. The prevalence of HT above this point was 14.5 % and much higher than that below the cutoff point (4.6 %). Similarly, ROC curve revealed a cutoff value of 2.6 mU/L, by which we can effectively detect HT with the best sensitivity and specificity.

These two measures showed very close results which are more close to the proposal in recent NACB guideline about narrowing the upper limit of TSH to 2.5 mU/L [11]. As proposed by Surks [16], the normal range for TSH should meet the needs to categorize population into groups 1) normal, 2) those who need to be observed closely and 3) those who need medication. Although the lowered cutoff value of TSH may not be used as a treatment threshold, it can help to find more patients with occult subclinical thyroid diseases. If the limit was increased to 4.5U/L, 2.2 % of individuals with HT would be missed.

It has been indicated by several studies [17–19], that elevated TSH within the normal range was positively related to lipid abnormalities or even to the future occurring of hypertension and hyperlipidemia. Likewise, we used the lowered cutoff value of TSH to categorize population to watch if it made sense concerning the metabolism parameters among groups. As expected, people in the group with higher TSH had a much higher age, higher rate of hyperlipidemia and a higher Framingham score. When the cutoff value of 2.6 mU/L was used, it showed better results with more significant difference between groups concerning hyperlipidemia (hyper-TC, hyper-LDL and hyper-TG). Meanwhile, if a lowered upper limit of TSH was used, we can detect more patients with hyperlipidemia with less missed diagnosis. The Framingham score went higher with the increasing of TSH. This was also consistent with Asvold’s observation [9] and suggested increased incidence of coronary heart disease in population with SCH. Therefore, a lowered upper limit of TSH can also help to detect individuals with increased risk of ischemic heart diseases effectively.

Certainly, there were several limitations in our study, including the higher proportion of females in the research population leading to a bias of TSH value which may be caused by the higher prevalence of HT in females. Secondly, the age of the research population was no older than 60, so the conclusion may not be applied to older people.

## Conclusion

This study shows a high prevalence of HT occurred among individuals with a TSH of 2.6–2.9 mU/L. These values are possible to the “true” values of normal upper limit of TSH for Chinese population. From the trend of higher abnormalities in metabolism-related indicators and Framingham score with the elevation of TSH, a proper cutoff value of normal upper limit of TSH will be useful in patients with mild thyroid dysfunction.
